# Medical care of acute myocardial infarction patients in a resource limiting country, Trinidad: a cross-sectional retrospective study

**DOI:** 10.1186/s12913-019-4344-2

**Published:** 2019-07-18

**Authors:** Mandreker Bahall, Terrence Seemungal, Katija Khan, George Legall

**Affiliations:** 1 School of Medicine, University of the West Indies, St. Augustine Campus, Trinidad and Tobago; 2 Department of Clinical Medical Sciences, University of the West Indies, St. Augustine Campus, Trinidad and Tobago; 3 Department of Clinical Medical Sciences, Psychiatry Unit, University of the West Indies, St. Augustine Campus, Trinidad and Tobago; 4 Department of Food Production and Agriculture, University of the West Indies, St. Augustine Campus, Trinidad and Tobago

**Keywords:** Acute myocardial infarction, Thrombolysis, Health care, Quality of care

## Abstract

**Background:**

Cardiovascular disease remains the most common cause of death. However, effective and timely secondary care contributes to improved quality of life, decreased morbidity and mortality. This study analyzed the medical care of patients in a resource limiting country with a first presentation of acute myocardial infarction (AMI).

**Methods:**

A cross-sectional retrospective study was conducted on first time AMI patients admitted between March 1st 2011 and March 31st 2015 to the only tertiary public hospital in a resource limiting country, Trinidad. Relevant data were obtained from all confirmed AMI patients.

**Results:**

Data were obtained from 1106 AMI patients who were predominantly male and of Indo Trinidadian descent. Emergency treatment included aspirin (97.2%), clopidogrel (97.2%), heparin (81.3%) and thrombolysis (70.5% of 505 patients with ST elevation MI), but none of the patients had primary angioplasty. Thrombolysis was higher among younger patients and in men. There were no differences in age, sex, and ethnicity in all other treatments. Of the 360 patients with recorded times, 41.1% arrived at the hospital within 4 h. The proportion of patients receiving thrombolysis (door to needle time) within 30 min was 57.5%. In-patient treatment medication included: aspirin (87.1%), clopidogrel (87.2%), beta blockers (76.5%), ACEI (72.9%), heparin (80.6%), and simvastatin (82.5%). Documentation of risk stratification, use of angiogram and surgical intervention, initiation of cardiac rehabilitation (CR), and information on behavioral changes were rare. Electrocardiogram (ECG) and cardiac enzyme tests were universally performed, while echocardiogram was performed in 57.1% of patients and exercise stress test was performed occasionally. Discharge treatment was limited to medication and referrals for investigations. Few patients were given lifestyle and activity advice and referred for CR. The in-hospital death rate was 6.5%. There was a significantly higher relative risk of in-hospital death for non-use of aspirin, clopidogrel, simvastatin, beta blockers, and heparin, but not ACE inhibitors and nitrates.

**Conclusions:**

Medication usage was high among AMI patients. However, there was very minimal use of non-pharmacological measures. No differences were found in prescribed medication by age, sex, or ethnicity, with the exception of thrombolysis.

## Background

Cardiovascular disease (CVD) remains the leading cause of morbidity and mortality worldwide [1–3]. Public health issues and primary health care are major factors in the development of coronary artery disease (CAD). Secondary prevention following acute myocardial infarction (AMI), with appropriate and timely treatment using evidence-based guidelines (EBG), such as those from the American College of Cardiology/American Heart Association (ACC/AHA) [4, 5], European Society of Cardiology (ESC) [6, 7], and National Institute for Health and Clinical Excellence (NICE) [8–10] improves survival [11], quality of life [11], quality of care [12], and patient satisfaction [13]. However, 26% of the opportunities for using EBG are missed [14]. According to the ACC/AHA, quality of care continues to lag behind expectations for organizational, as well as individual patient care [15]. This may be further worsened in a resource limiting country. A study of American Indians revealed the use of aspirin on admission and at discharge, reperfusion therapy within 24 h, prescription of beta blockers at discharge, and smoking cessation counseling were 94, 91, 92, 86, and 71%, respectively [16]. Another study reported a high rate of patients not being given basic medications such as aspirin within 24 h of admission (17.0 to 23.6%) and beta-blockers on arrival and at discharge (30.8 to 46.6%) [17]. In a study by Maharaj et al. [18], only 20.5% of patients with ST elevation MI (STEMI) met the ACC benchmark of receiving fibrinolytics with a door-to-needle time of 30 min or less.

Treatment gaps in resource limited developing countries may be worse. Emergency management of AMI with EBG medications such as aspirin, clopidogrel, anticoagulants, and thrombolytics for STEMI, and inpatient management with medication, risk assessment, surgical intervention, and patient education, advice on daily activities and lifestyles, and initiation of cardiac rehabilitation (CR) may fall short of expectations. In this study we analyzed the medical care of first time AMI patients at a public tertiary health care institution in a resource limiting, small developing country. The study aimed to describe the medical care of, and outcomes of AMI patients in a resource limiting country. This will assist health care providers in identifying gaps in care and reshaping patient management to enhance care and ultimately improve clinical outcomes.

## Methods

### Study region

The study was conducted at the San Fernando General Hospital (SFGH), the only free tertiary public health institution in south Trinidad. The SFGH provides care to approximately 600,000 patients per year. Annually, there are 46,785 admissions including 15,339 medical admissions (2010) [19]. The incidence of AMI is 90.6 per 100,000 [20].

### Setting

The SFGH has a 25-bed cardiac unit managed by approximately 18 registered nurses, and 8 (2011) to 19 (2017) doctors, two of whom are non-invasive cardiologists. The unit’s facilities are limited to bedside electrocardiogram (ECG) monitors (about 11), cardiac resuscitation carts and facilities for temporary pacing and portable echocardiography. More difficult cases that require ventilation are managed in the general intensive care unit (ICU) of the hospital. The institution has significant resource constraints and an inconsistent supply of useful diagnostic tests and services. There is no chest pain unit and no facilities for primary angioplasty or other cardiac surgical interventions.

### Sampling and data collection

The study frame included all patients admitted with a diagnosis of AMI according to the ACC/AHA guidelines definition. AMI is defined as evidence of myocardial cell necrosis due to significant and sustained ischemia [21]. It is defined clinically as a rise and/or fall of cardiac biomarkers with at least one of the following: symptoms of ischemia, and ECG changes indicating ischemia, echocardiographic or angiogram findings indicating ischemia [22].

All patients admitted with AMI between March 1, 2011 and March 31, 2015 were included in the study. There were no exclusion criteria; however, doubtful cases and cases treated as AMI that did not fulfill the definition criteria were excluded. Confirmed cases of AMI that could not be clearly identified as STEMI or non-STEMI (NSTEMI) were named as unclassified and included cases with missing, non-interpretable or disputed ECGs. The files of patients with a discharge diagnosis of AMI were reviewed, and confirmed cases of AMI were selected for the study.

Data was collected by research assistants and corroborated with clinicians. The chief investigator supervised and assisted in data collection and interpretation. Research assistants included 4 premedical students, a medical doctor, and two post graduate students, all of whom were trained in data collection from medical records and the working of the medical institute. All of them were officially assigned to the researcher for this project. All data were collected via a questionnaire. We extracted data on general patient information (patient characteristics, lifestyle, weekly exercise frequency, and self-reported level of stress), medical history (diabetes mellitus [DM], hypertension, hypercholesterolemia, ischemic heart disease (IHD), renal insufficiency, or any other relevant medical problems), social and family history (smoking, alcohol, cocaine and marijuana, and family history of IHD), presenting symptoms, selected lab data, ECG, other investigations (echocardiogram, angiogram, and exercise stress test), and complications. Data also included emergency department (ED) clinical data (symptoms, heart rate, systolic blood pressure [SBP] and diastolic blood pressure [DBP]), treatment (analgesia, aspirin, clopidogrel, beta blocker, heparin, thrombolytics, and surgical intervention), and relevant recorded times (time from onset of chest pain to hospital and time from hospital arrival to thrombolytic therapy). Data collected were entered in a computer which was accessible to the researcher and research assistants only.

### Analysis and interpretation

Data were analyzed using descriptive and inferential statistics. Multivariate binary logistic regression was also used to identify the predictors of the likelihood of receiving thrombolytic therapy. Analysis of variance methods were used for comparing means for continuous variables, and the chi-square test was used for testing the association between selected pairs of categorical variables and to compare frequencies. The significance level was set at *p* < 0.05, while the limit for trends was set at *p* < 0.10.

Ethical approval was granted by the Ethics committee of the SWRHA and the University of the West Indies.

## Results

A total of 1134 eligible AMI patients were identified from patient registration records. Usable data were obtained from 1106 (97.5%) patients who were identified as fulfilling the criteria for AMI in the ED of the hospital. Patient characteristics are presented in Table 1. Patients were predominantly male and of Indo-Trinbagonian descent.

**Table 1:** Patient characteristics and presenting symptoms

Description	N	%
*Patient characteristics*		
Sex^a^		
Male	744	67.3
Female	357	32.3
Age group (years)		
<45	159	14.4
45–64	551	49.8
≥65	396	35.8
Ethnicity		
Afro-Trinidadian	180	16.3
Indo-Trinidadian	860	77.8
Mixed-Trinidadian	64	5.8
White Trinidadian	2	0.2
AMI		
STEMI	505	45.6
NSTEMI	545	49.3
Non-classified^b^	56	5.1
Clinical factors		
Diabetes	626	56.6
Hypertension	731	66.1
Hypercholesterolemia	165	14.9
Ischemic heart disease	504	45.6
Renal insufficiency	206	18.6
Other health condition	196	17.7
Lifestyle Factors		
Current smoker	380	34.4
Ex-smoker	61	5.5
Use alcohol	348	31.0
Use cocaine	43	3.9
Use marijuana	19	1.7
Presenting symptom		
Classic chest pain	800	72.3
Palpitations	235	21.2
Sweating	284	25.7
Dizziness	257	23.2
Shortness of breath	428	38.7
Nausea	266	24.0
Vomiting	129	11.7
Diaphoresis	226	20.4
Atypical chest pain	91	8.3

^a^missing data for 5 patients

^b^Difficulty in ECG interpretation as STEMI or missing ECG

The overall mean (standard deviation [SD]) patient age was 58.6 ± 13.43 years with the mean age of the women higher than that of the men (62.2 ± 13.30 years vs 56.9 ± 13.15 years, respectively, *p* ≤ 0.001). Hypertension was the most prevalent traditional risk factor followed by DM, IHD and renal insufficiency. The most common lifestyle factor was being a smoker or ex-smoker followed by use of alcohol, use of cocaine, and use of marijuana. The most common presenting symptom was classic chest pain (*n* = 800, 72.3%) (Table 1). The mean HR (SD) was 85.2 (31.28), the mean SBP (SD) was 147.9 (32.29) and the mean DBP (SD) was 88.0 (19.76).

The two main diagnostic tools used in the ED were ECG, performed on all patients, and troponin levels, which were performed on 744 (67.3%) of the patients. ECG findings on arrival to the ED were as follows: sinus rhythm (74.2%), ventricular tachycardia (0.6%), supraventricular tachycardia (0.0%), atrial fibrillation (12.5%), unequivocal ST elevation (44.4%), ST depression (24.9%), and other unspecified findings (46.6%).

Desired emergency management included aspirin, clopidogrel, and heparin for all patients as well as thrombolysis for STEMI patients. Primary angioplasty was unavailable. Other treatments administered were analgesia, oxygen, nitrates and beta-blockers if indicated. The most common emergency treatments were aspirin and clopidogrel (97.2%), followed by heparin (81.3%) (Table 2). A few patients received aspirin and/or clopidogrel before arrival at SFGH; either through self-medication or provided by emergency services providers. Thrombolysis remains the standard treatment for STEMI patients at public health institutions in Trinidad and Tobago.

**Table 2:** Emergency treatment

	Total patients excluding missing data, % (proportion)	
	Proportion	%
*Mandatory (EBG)*		
Aspirin (*n* = 790)	768	97.2
Clopidogrel (n = 792)	770	97.2
Enoxaparin (Heparin) (*n* = 792)	644	81.3
Thrombolysis *for STEMI patients*(*n* = 505) *if no contraindication*	356	70.5
*If necessary*		
Oxygen (*n* = 791)	152	19.2
Analgesia (n = 791)	723	91.4
Beta blocker (n = 792)	240	30.3
Nitrates (*n* = 759)	114	15.0

There was no association of age and sex with any of the core treatments except for thrombolysis. The use of thrombolysis was significantly higher in men than in women; and in younger compared to older patients. Chi-squared analysis showed a significant association between receiving thrombolytic therapy and sex (*p* ≤ 0.001); age (*p* ≤ 0.001); waist circumference (*p* ≤ 0.001); time from onset to arrival at the hospital (*p* ≤ 0.001); and presence of DM (*p* ≤ 0.001), IHD (*p* ≤ 0.001), and renal insufficiency (*p* ≤ 0.001). Furthermore, multivariate binary logistic regression revealed that only waist circumference (OR, 0.539; 95% CI, 0.352–0.826; *p* = 0.005) and patient pre-hospital delay time (time from home to hospital) (OR, 0.767; 95% CI 0.625–0.941; *p* = 0.010) were useful predictors for the likelihood of receiving thrombolytic therapy.

**Fig. 1:**
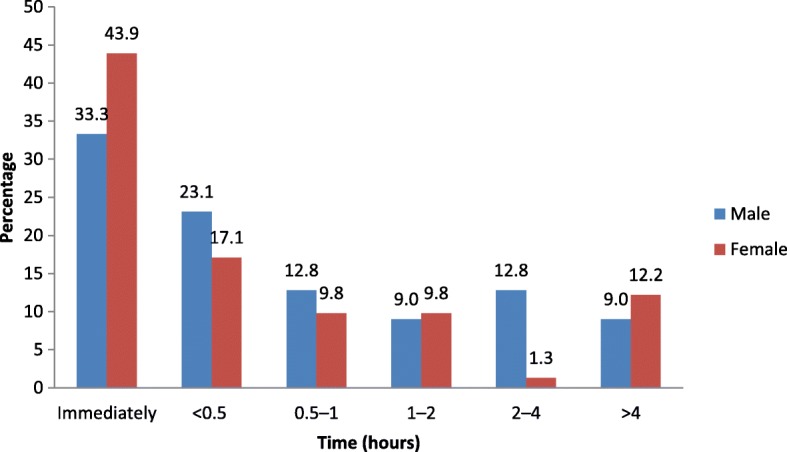
Time from hospital arrival to administration of thrombolysis

Time from home to hospital was not recorded for the majority of patients. However, of the data available for 360 patients, the time ranged from less than 4 h (41.1%) to over 24 h (27.8%). 17.8%, 7.8 and 5.6% of patients arrived within 4–8 h, 8–12 h and 12–24 h respectively. The time variation among men (238/745, 31.9%) and women (121/358, 33.8%) was not significantly different (*p* = 0.537). 41.6% males and 40.5% females took less than 4 h from symptom onset to hospital arrival, 19.7% males and 14.0% females took 4–8 h and 25.2% males and 33.1% females took over 24 h. The minority, (Males: 8.0%, Females: 7.4%) and (Males: 5.5%, Females: 5.0%) had ranges of 8–12 h and 12–24 h respectively. Information regarding time from arrival at hospital to receiving thrombolytic therapy was available for 120 patients; this showed that the majority received thrombolysis within 30 min of arrival at the ED (Fig. 1). Further analysis showed no association between time from arrival at the ED and receiving thrombolysis and sex, age, or ethnicity. Data regarding ward assignment (medical, cardiac, or ICU) of the patients and length of stay in the ED were generally not well documented.

**Fig. 2:**
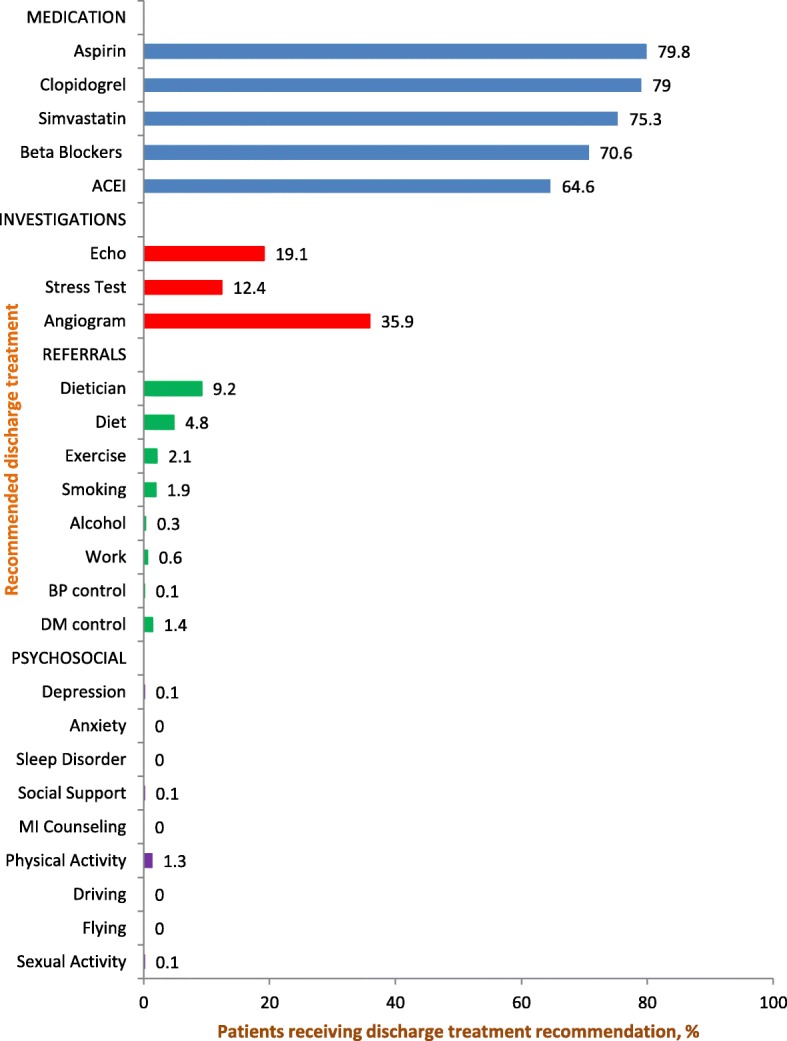
Frequency of discharge treatment recommendation. (Legend) ACEi, angiotensin converting enzyme inhibitor; BP, blood pressure; DM, diabetes mellitus; MI, myocardial infarction

Admitted patients to the ward (medical or cardiac ward) were treated with aspirin (87.1%), beta blockers (76.5%), clopidogrel (87.2%), statins (82.5%) and angiotensin-converting enzyme inhibitors (ACEi) (72.9%). No significant differences were found in age group, sex, and ethnicity for any of these drugs (Table 3). Very rarely was percutaneous coronary intervention (PCI) or coronary artery bypass grafting (CABG) performed while as an inpatient. If these procedures were not done as an inpatient, recommendations were made following discharge. Discharge plans were limited to medication (63.0% for nitrates, 64.6% for ACEI, 70.6% for beta blockers, 75.3% for simvastatin, 79.0% for clopidogrel and 79.8% for aspirin) and referrals for investigations with little lifestyle advice, medical counseling, daily activities advice, and referral for cardiac rehabilitation (Fig. 2).

**Table 3:** Hospital treatment

Indicator	EBG Treatment (commencement)	ED treatment, % use in study	In-patient treatment, % use in study
Drugs	Dose (commencement)		
Aspirin	300 mg then 81 mg daily (Day 1)	97.2	963(87.1)
Clopidogrel	300 mg then 75 mg daily (Day 1)	97.2	964(87.2)
Enoxaparin (Heparin)	1 mg/kg twice daily (Day 1)	81.3	80.6
ACE inhibitor/ARB	Initiate with low dose and titrate upwards (Day 1)	NA	806(72.9)
Beta blocker	Initiate with low dose and titrate upwards (Day 1)	30.3	846(76.5)
Statin	Night time max tolerated dose (Day 1)	NA	913(82.5)
Primary PCI	Not applicable (not a PCI center)	NA	NA
Nitrates	As required	15.0	809(73.1)
Investigations			
ECG	Daily (Day 1) at least 2	100.0	NA
Cardiac enzymes	Daily (Day 1) at least 2	67.3	1106(100)
Echocardiogram	Prior to discharge	NA	632(57.1)
EST	If necessary	NA	NA
Risk stratification			
TIMI or GRACE score or other	Within 24 h	NA	NA
Surgical intervention			
Early invasive NSTEMI	Intermediate- and high-risk patient: 24 to 48 h of admission	Nil	Nil
Late invasive NSTEMI	Low-risk patient: 25 to 72 h of admission	Nil	Nil
Lifestyle counseling			
Diet	Hospital stay	Nil	4.8
Smoking	Hospital stay	Nil	1.9
Exercise	Hospital stay	Nil	1.3
Daily activity	Hospital stay	Nil	0.0–0.6
Cardiac rehab initiated	Hospital stay	Nil	0.0
Discharge medication	Pharmacist review day 4 and before discharge	Nil	Nil

*ACE* angiotensin-converting enzyme, *ARB* angiotensin II receptor blocker, *ECG* electrocardiogram, *EST* exercise stress test, *GRACE* Global Registry of Acute Coronary Events, *HR* heart rate, *NSTEMI* non-ST-elevation myocardial infarction, *PCI* percutaneous coronary intervention, *STEMI* ST-elevation myocardial infarction, *TIMI* Thrombolysis in Myocardial Infarction, *NA* Treatment not available or missing data

## Outcomes

The average length of stay was 6.71 ± 5.77 days (range, 1–61 days). The majority of patients stayed between 3 and 8 days (*n* = 756, 70.9%) while a minority stayed < 3 or > 8 days (*n* = 311, 29.1%). Less than 2 % of the patients suffered at least one of the following complications: hypotension, arrhythmia, and bradyarrythmia requiring temporary pacing, recurrent ischemia or infarction, left ventricle failure, pericarditis, bleeding requiring transfusion, infection, and new neurologic event. Of the 1106 patients in the sample, 72 in-hospital deaths were recorded, which equates to an in-hospital death rate of 65 per thousand (46 male and 26 female, which is equivalent to an in-hospital death rate of 6.18 and 7.2% for males and females, respectively).

## Discussion

In this resource limiting country, AMI treatment largely focused on pharmacological treatment. EBG emergency treatment comprising of aspirin (97.2%), clopidogrel (97.2%), and heparin (81.3%) was relatively high. Thrombolytic treatment was received by the majority (70.5%) of patients. The use of thrombolysis was significantly higher in men than in women; and in younger compared to older patients. The proportion of patients thrombolysed (*n* = 356/505, 70.5%) compares well or is even better than in other developing countries. In Sri Lanka, 70.2% of STEMI patients receive thrombolysis [23], 41% of STEMI patients in India [24], 44.7% in Cape Town [18], 59% in Iran [25], 62% in Kenya [26] and 27% at a tertiary-care hospital in Sri Lanka [27]. Our study also compares well with studies from first world countries such as Scotland [28]. A study done locally at the Eric Williams Medical Sciences Complex in Trinidad in 2008 found that 78.4% of STEMI patients received thrombolytic therapy [29]. The significantly higher percentage of thrombolysis done in men and younger patients is a cause for concern since there is no policy to favour these groups. It may be because of earlier recognition of AMI and less distraction to reach hospital.

In our study, of the 120 STEMI patients with available treatment time data, 57.5% received thrombolysis within 30 min. There was no association between time from arrival at the ED and receiving thrombolysis with sex, age, or ethnicity. EBG for emergency medical care (triaging, ECG acquisition time, door to thrombolysis time) of AMI is well recognized [30–32]. Reperfusion within the golden hour may abort 25% of AMIs [33]. The GUSTO trial revealed that only 7.3% of patients were treated within the first 30 min [34]. Timely interventions must be achieved to decrease coronary artery thrombus formation and prevent extension of existing thrombus, regardless of the type of intervention [35]. In fact, the risk of 1-year mortality is increased by 7.5% for each 30-min delay [35]. Although primary PCI is the superior treatment option, the value of thrombolysis should not be underestimated. A study by Armstrong et al. [36] reported no significant difference in primary composite outcome (death, shock, heart failure, or re-infarction at 30 days) between early thrombolysis and PCI [36]. Thrombolysis is a well-recognized treatment when angioplasty is unavailable. Timely reperfusion through thrombolytic therapy or angioplasty [5] improves outcome by decreasing infarct size [37], and lowers morbidity and mortality [38, 39].

Patient delay (symptom onset to hospital arrival) was quite high with 58.9% arriving more than 4 h after symptom onset and more than 25% arriving after 24 h; therefore, only a few patients benefitted from being treated within the golden hour. This may result from lack of resources and cultural challenges; unclear patient and health care provider policies to ensure early recognition, patient decision and swift transport to appropriate medical care. Delayed treatment due to patient procrastination has been reported in numerous studies: 58% more than 2 h [26], 49.5% more than 4 h [40], 40% more than 6 h [41], and 80.9% more than 12 h [42].

In-hospital patient treatment in our study consisting of aspirin (87.1%), beta blockers (76.5%), ACEI (72.9%), statins (82.5%), and nitrates excluding glyceryl trinitrate (73.1%) compares well with other studies [43, 44]. ACEI usage was higher in our study than that of Callender et al. [45] who found that 57% of patients were treated with ACEI. Statin usage in our study was also higher than the 61% cited by Rasmussen et al. [46] in their study of first time AMI patients in Denmark. In our study, no significant difference in medication usage by age, sex or ethnicity was found for in-patient care. These auger well for our country in terms of the absence of systemic discriminatory practices in its largely pharmacological focus. This is in contrast with other studies, where significant differences in treatment among patients of different ages and sex were found [47–49].

The two most basic investigations, serial ECGs and troponin levels, were obtained on all patients once admitted. Some admitted patients may have gotten their troponin levels done at private labs. ECG monitoring, however, is performed in fewer patients and is not documented consistently. Echocardiograms were performed in 632 (57.1%) patients. In-patient echocardiography is important to identify high-risk patients with a poor ejection fraction or patients who may develop complications such as left ventricular thrombus and cardiac and papillary muscle rupture [50].

In our study, risk evaluation was rarely done, despite the importance of this assessment in order to identify high and intermediate-risk patients who would benefit from early or delayed in-patient surgical intervention or who may be at higher risk of mortality [51]. Low-risk patients, if identified, may benefit from nonsurgical treatment, which may be as effective as surgical intervention [52]. It is possible that the unavailability of early invasive surgical intervention may be the rationale for not routinely doing risk assessments.

Nearly all patients were confined to their beds during their hospital stay because of the unavailability of bedside space. Ideally, patients should sit out of bed, use a commode and undertake self-feeding and self-care after 12–24 h, if free of recurrent ischemic discomfort, symptoms of heart failure, or serious arrhythmias. Assisted ambulation, where the patient can walk up to 300 m on a flat surface, should be commenced the following day. Those whose infarctions are complicated by heart failure or serious arrhythmias should be kept in bed longer and their physical activity increased slowly [53].

Discharge medications included nitrates (63.0%), ACEI (64.6%), beta blockers (70.6%), simvastatin (75.3%), clopidogrel (79.0%) and aspirin (79.8%). The patients discharge treatment excluded evidence of medicine reconciliation i.e. where health care professionals partner with patients to ensure an accurate and complete transfer of medication information at interfaces of care. Other dimensions of care in discharge treatment package should include diagnosis and prognosis information, lifestyle advice, comorbidities management, risk factor modification, counseling for psychosocial issues, clinic referrals and cardiac rehabilitation [4, 6, 54–57].

However, few patients in this study received information, advice, or counseling on smoking (1.9%), diet (4.8%), physical activity (1.3%), and daily activities (driving, sexual activity, air travel, and return to work) (0.0–0.6%). This is notable since studies have demonstrated that smoking cessation reduced the subsequent cardiovascular mortality rate by nearly 50% [58]. Exercising (walking, jogging, cycling, or other aerobic activity) for a minimum of 30 min, preferably daily, but at least 3 or 4 times per week supplemented by an increase in daily lifestyle activities (e.g., walking breaks at work, gardening, and household work) [59] should also be encouraged. Appropriate diet [60] decreases CVD risk. Cardiac rehabilitation, comprising nutritional counseling, risk factor control [60], psychosocial and physical activity counseling, exercise training, and pharmacological treatment [61] improves secondary prevention and increases functional capacity, decreases or alleviates angina symptoms, reduces disability, improves quality of life, modifies coronary risk factors, and reduces morbidity and mortality rates [62]. Cardiac rehabilitation must be initiated before discharge and continued post discharge [61, 63].

The importance of risk factor control, lifestyle changes and appropriate daily activities has been widely discussed. Risk factor modification must be recommended to ensure risk factor targets are achieved [11, 64, 65]. EBG recommend strict BP control with a target of < 140/90 mmHg [6] or less than 130/80 mmHg for persons with DM or CKD [66], glycosylated hemoglobin levels to < 7% [67] for diabetic patients, LDL < 100 but preferably < 70 mg/dl is recommended [67] for hyperlipidemia, psychiatry referral for major depression [68–70] and a BMI target of 18.5 to 24.9 kg per m^2^ [71] with appropriate dietary and weight management advice and support [72–75]. It is recommended that daily alcohol intake be limited to 1 unit and 2 units daily for women and men, respectively, and if possible, should be avoided [76]. Recommended activities of daily living will depend on individual symptoms. Activities of daily living include air travel for patients without symptoms of angina, dyspnea, or hypoxia at rest, 2 weeks after AMI [77] and sexual activity within 1 week to 10 days [78]. Low risk patients who received medical treatment and revascularization should be followed up in 2–6 weeks, while higher risk patients should be reviewed within 14 days. Relevant investigations requested should include an echocardiogram [79], EST [80] and angiogram [81].

The average length of stay of 6.71 ± 5.77 days (range, 1–61 days) is higher than 5.3 days obtained for Kenya [26], a developing country. Developed countries which are able to provide additional care have a higher average length of stay: 11.9 days in 2011 for China [82], 9.9 days in Canada [83], and 8.7 days in 2009 for Germany [84]. The prolonged length of stay for patients in our center, which were not provided with surgical intervention, cardiac rehabilitation or behavioral treatment, may be due to the lack of resources and inability to obtain essential investigations such as echocardiography; waiting for an inpatient bed; or delayed discharge due to patients awaiting medication or relatives to take them home. Complication rates affected less than 2 % of the patients. Of the 1106 patients in the sample, 72 in-hospital deaths were recorded, which equates to an in-hospital death rate of 65 per thousand. There were 46 male and 26 female in-hospital deaths, resulting in a death rate of 6.18 and 7.2%, respectively. This contrasts with the findings of Jose et al. [85] who found the in-hospital mortality rate of acute STEMI in an Indian hospital to be 16.9%, which is approximately three times the overall death rate for AMI at our medical institution.

### Limitations

This study is a retrospective study that depended on previously recorded data; therefore, some cases of AMI might have been missed. Furthermore, there is a lack of or limited information in the patient records on height, weight, waist and hip measurements; lab data; times; and advice given. A lack of definitions on variables such as family history, history of IHD, and smoking may lead to over or underreporting. Missing data in the emergency records may result from omission at entry of prior medication usage, non-recorded contraindications, side effects or allergies, and failure to document items obtained from out of pocket spending. Lack of comprehensive documentation may have led to under-estimation of drug, lifestyle advice, and other medical counseling prescribed.

## Conclusions

Patient care has largely focused on pharmacological treatment with little or no emphasis on surgical intervention, medical counseling, lifestyle advice, activity advice, and cardiac rehabilitation. Patient factors contribute significantly to delayed treatment with close to 60% presenting to the ED after 4 h. However, institutional issues may be responsible for a significant percentage of STEMI patients (42.5%) who fail to access prompt treatment and thrombolysis within 30 min of arriving at the ED. In our study, with the exception of thrombolysis treatment, no treatments showed any differences in terms of sex, age, or ethnicity. Similar findings were obtained for in-hospital treatment. A proper emergency, inpatient, and discharge care package treatment is recommended. There is also a need to provide appropriate documentation in medical records (triage time, ECG acquisition time, and thrombolytic/PCI time) to facilitate feedback for quality health care. The unavailability of primary angioplasty should encourage a more aggressive and timely use of thrombolysis.

## Data Availability

The data that support the findings of this study are available from the corresponding author on request.

## Abbreviations

ACC/AHA: American College of Cardiology/American Heart Association
ACE: angiotensin-converting enzyme
AMI: acute myocardial Infarction
ARB: angiotensin II receptor blocker
CABG: coronary artery bypass grafting
CAD: coronary artery disease
CI: confidence interval
CVD: cardiovascular disease
EBG: evidence-based guidelines
ECG: electrocardiogram
ED: emergency department
EST: exercise stress test
IHD: ischemic heart disease
LAD: left anterior descending
LDL: low density lipoprotein
LV: left ventricular
LVEF: left ventricular ejection fraction
NSTEMI: non-ST-elevation myocardial infarction
OR: odds ratio
PCI: percutaneous coronary intervention
SD: standard deviation
SFGH: San Fernando General Hospital
STEMI: ST-elevation myocardial infarction
